# Formalization of the Burning Process of Virtual Reality Objects in Adaptive Training Complexes

**DOI:** 10.3390/jimaging7050086

**Published:** 2021-05-12

**Authors:** Mikhail Krasnyanskiy, Artem Obukhov, Denis Dedov

**Affiliations:** 1 Department of Administration, Tambov State Technical University, 392000 Tambov, Russia; mikhail.krasnyanskiy@yandex.ru; 2 Department of Automated Decision Support Systems, Tambov State Technical University, 392000 Tambov, Russia; 3 Department of Basic and Applied Research, Tambov State Technical University, 392000 Tambov, Russia; hammer68@mail.ru

**Keywords:** adaptive training complexes, formalization of burning process, ergatic systems, model of states of virtual objects

## Abstract

Within the scope of this article, the problem of the formalization of physical processes in adaptive training complexes is considered on the example of virtual objects burning. Despite a fairly complete study of this process, the existing mathematical models are not adapted for the application in training complexes, which leads to a significant increase in costs and lower productivity due to the complexity of the calculations. Therefore, an adapted mathematical model is proposed that allows us to formalize the structure of virtual objects of burning, their basic properties and the processes of changing states, starting from the flame development of an object and ending with their complete destruction or extinguishment. The article proposes the use of threshold value diagrams and rules for changing the states of virtual reality objects to solve the problem of the formalization of burning processes. This tool is quite multi-purpose, which allows you to describe various physical processes, such as smoke, flooding, the spread of toxic gases, etc. The area of the proposed formalization approach includes the design and implementation of physical processes in simulators and multimedia complexes using virtual and augmented reality. Thus, the presented scientific research can be used to formalize the physical processes in adaptive training complexes for professional ergatic systems.

## 1. Introduction

Currently, one of the main safety problems in industrial plants is the burning of a different class. According to the statistics of the Ministry of Emergency Situations of the Russian Federation, about three thousand fires in industrial buildings occurred in 2018. The main causes of burning are violation of the rules for the electrical equipment operation, violation of the safety rules for gas welding operations and careless handling of fire. Therefore, training the personnel of an industrial enterprise to properly assess the situation in the event of a fire and take measures to prevent dangerous consequences is an important and urgent task [1,2,3].

There are various ways to improve the level of personnel training, some are as follows: theoretical training, briefings, practical exercises, and conducting training evacuations and exercises; however, in recent years, adaptive training complexes (ATC) have shown an increasing efficiency [4]. They are complex software and hardware systems that allow training using advanced technologies of augmented and virtual reality, taking into account the psychological and physical characteristics of a person [5].

However, there are still unresolved issues and areas of interest in the sphere of the training complex’s design. This includes the task of formalizing the process of objects burning in virtual reality, which is considered in this article. Realistic and accurate modeling of the burning process is very important because it allows you to develop the right practical skills among students; however, it is pointless to completely transfer the physical processes of flame spread and material burning, and the effect of air flow, tightness and humidity. Firstly, it is impractical with respect to the final result, secondly, it will lead to a significant loss of productivity, and thirdly, it will significantly increase the cost of the training complex [6,7,8].

Existing models can predict fire propagation and smoke movement from an engineering point of view, but since these models are extremely computationally complex, this makes them difficult to apply in virtual reality systems. Some examples of such models and approaches are FDS, FireFOAM, SmartFire, ANSYS FLUENT, CFX, etc. [9,10,11]. For example, the Smokeview software is currently used by engineers to visualize various hydrodynamic and physical processes [12].

Therefore, the crucial task is to replace the mathematical model of the physical burning process with the simplified one, developed and adapted for virtual reality. The obtained model should, on the one hand, be distinguished by its simplicity of implementation, speed, be easily reproducible in the program code, and on the other hand, it must meet the necessary requirements for realism and correspond to the actual burning processes with sufficient accuracy.

## 2. Materials and Methods

In order to solve the stated problem of formalizing the burning process, an appropriate mathematical apparatus is used, which allows describing in sufficient detail the main objects and their interaction within the subject domain.

Analysis of existing approaches to modeling the combustion process has shown the following results.

The following three main groups of deterministic models are used to describe the parameters of a fire: integral, zone (zonal), and differential (field) [13,14,15]. The models differ from each other in the amount of information they can give about the state of the gaseous environment in the room and the elements interacting with it at different stages of the fire. Mathematically, these fire models are characterized by different levels of complexity.

The integral model of fire is represented by a system of ordinary differential equations describing the change in the volume average parameters of the state of the gaseous environment in a room during the development of a fire. The sought-for functions are the volume average parameters of the state of the environment, time is an independent argument.

In the general case, the zone model of fire is based on a combination of several systems of ordinary differential equations. The parameters of the state of the environment in each zone are the required functions, and the independent argument is time. Coordinates that determine the position of the boundaries of characteristic zones are also the sought-for functions.

The most difficult in mathematical terms is the differential (field) model. It is based on a system of partial differential equations describing the spatio-temporal distribution of temperatures and velocities of a gaseous medium in a room, the concentrations of the components of this medium (oxygen, carbon monoxide, and dioxide, etc.), pressures, and densities. These equations include Stokes’ rheological law, Fourier’s law of thermal conductivity, laws of diffusion and radiative transfer, etc. In the general case, a differential equation of thermal conductivity is added to this system of equations, which describes the process of heating the enclosing structures. Such computer programs as PYROSIM, CFX, FDS, ANSYS FLUENT [9,10,11] may be cited as an example of the numerical implementation of differential models that describe the fields of velocities, temperatures, and concentrations at the initial stage of a fire fairly accurately.

The use of computer programs for solving practical problems of the dynamic development of fires, especially those that implement differential mathematical models, requires a significant investment of time and machine resources to obtain and adequately interpret the results. Besides, for many applied issues of fire technical problems in visualization tools, such detailed information about the dynamics of the fire is not required, as long as differential models can provide it. Usually, using a computer program to study the dynamics of a fire is a laborious procedure for most engineering calculations, especially due to the need to analyze a multifactorial problem.

Thus, we have the following disadvantages of the existing deterministic models: high complexity of integration into virtual reality systems and low performance. Existing studies have shown that when calculating the combustion of one object even, the number of frames drops to 30 on sufficiently powerful equipment [16], which makes this approach inapplicable for many simultaneously burning objects. In [17], 4 Quadro K5000 video cards were used (total performance of over 8.5 gflops). On this equipment, about 10 frames were achieved when rendering 10 scenes with CFD (Computational Fluid Dynamics), which also cannot be considered satisfactory for VR (Virtual Reality).

The analysis of approaches to the formalization of physical processes of burning and methods for their modeling in training complexes allowed us to choose the mathematical apparatus of a set theory used to formalize the main objects and processes of burning, as well as their structure, which will correspond to classes and attributes in the object-oriented paradigm of programming languages [8,18,19,20].

**Figure 1:**
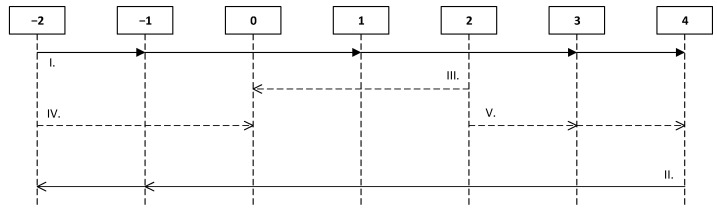
Example of virtual object life cycle diagram. Source: developed by the authors.

It is necessary to use graphical representations (life cycle diagrams) based on graph theory and project planning methods, which are more visual to describe the life cycle of virtual objects in the process of their flame formation, burning, extinguishing, or complete destruction. These diagrams show the life cycle of each virtual object. Vertical lines (from −2 to 4) are the threshold states of the combustion object. Horizontal lines with directional arrows represent the life cycle of the object transitions between states. Figure 1 shows separate unrelated fragments of the life cycle of an object with typical transitions between states, which will be described in detail below. Real life cycles will be discussed in the results section.

Thus, the life cycle diagram reflects a change in the threshold values of an object within one event over time, if you move along the horizontal axis from left to right, and the transition of an object from one event to another in accordance with the state transition rule system, if you move from top to bottom.

Threshold values are central to the modeling of the entire burning process. The value of this parameter directly depends on all external influences on the object and its own properties of the object (for example, temperature) and affects the calculations of the burning rate and, as a result, the factor of safety. This parameter changes during the life of the object and affects what event can occur with the object or what effect can be exerted on the object at a given time (and receives a response).

Some threshold states of the object may also be absent due to the type of material, for example, gasoline, which when exposed to open fire ignites immediately, i.e., initial threshold states will be skipped (“1”, “2” and “3”). Therefore, the list of these values is indicated individually for all materials. They can be absent due to the nature of the material.

The event means the occurrence, end or change of any influence on objects involved in the burning process. Events can occur both when performing combinations of certain conditions, states or environmental influences (including human actions, such as using water), as well as the object itself, leading to a change of state, changing parameters or other consequences that affect the burning process in some way (type of material that provides the possibility or impossibility of flame formation).

The diagram of threshold values is based on the results of the analysis of fire hazardous properties of materials, mechanisms for the occurrence and spread of fires, as well as their liquidation when using fire extinguishing agents [19,20]. The main values of the threshold values that we used in the formalization of the burning process are presented in Table 1.

The second important part of the diagram is the rules for changing states, marked on it by Roman numbers (Figure 1) and reflecting all possible transition states of the object. All the rules describe the actual physical processes occurring in objects with a particular type of influence or its absence, as follows:

- Rule 1—this rule describes the process of changing the state of an object with an external effect positive for the burning process (for example, a spark or fire). A prerequisite for compliance with this rule is the continuity of this positive impact. The strength of the object begins to decline only after reaching the state “2”;
- Rule 2—it describes the process of changing the state of an object when an external influence is negative for the burning process (for example, water or sand). As in the case of the first rule, the condition for its observance is the continuity of the negative impact. During the impact, the strength of the object can gradually decrease (until the state reaches “0” state);
- Rule 3—this rule is responsible for the process of returning the object to the neutral state “0”. An example of this process can serve as an unsuccessful attempt to set a fire. The main condition for the fulfillment of this rule is the termination of a positive impact within the threshold values from “0” to “2”;
- Rule 4—also, as in the previous rule, it describes the transition of an object to its neutral state “0”, with the exception that this rule describes, for example, the drying of an object after water extinction. The main condition for the implementation of the rule is the stop of negative impact within the threshold values from “−2” to “−1”;
- Rule 5—it describes the process of an object’s tendency for burning, even after the end of a positive effect. The condition for the execution of the rule is the termination of the positive impact within the threshold values from “2” to “4”.

The influences on virtual objects can be divided into the following two types: with a positive effect for the burning process (fire, sparks or temperature) and negative (water, foam, sand, powder, or CO_2_). Thus, positive effects affect the development of the burning process, and negative ones lead to its termination, which is also reflected in the change in the threshold value at the corresponding direction.

For example, fire contributes to the spread of the flame to those objects with which it interacts, and has a temperature effect on objects nearby (even without direct contact with the flame). The sparks mean a molten metal during welding, cutting, etc., which during contact with an object has a high-temperature effect, leading to fire.

Water is used to extinguish and cool objects and has an obstructive effect on flame formation and spread. It is also possible to use various types of fire extinguishers (foam, powder and carbon dioxide) to extinguish objects, thereby having a negative effect on the burning process. Sand is very effective where water no longer has a large effect (the extinguishing of electrical wiring, pools of flammable substances, etc.).

Any type of influence has a different power depending on the type of source of this impact, and the material of the object.

Some of the following environmental conditions can also be referred to as influences:

1. The volume of the virtual room;
2. Circulation of air in the room with turned on room ventilation affects the direction of fire propagation. Ventilation can be natural, mechanical and combined;
3. Switched on electricity can affect the short circuit of electrical appliances when water enters and other consequences associated with electricity in the event of a fire and an attempt to eliminate it;
4. The availability of oxygen and the tightness of a room affect the burning force of objects (the height of flame, the speed of propagation, etc.). Objects may stop burning or not flame up at all without enough oxygen;
5. The temperature of the room affects the initial state of the objects and depends on the amount of natural heat sources for this room and the fire sources, as well as on whether ventilation is turned on;
6. Gas hazard and smoke in a room is completely dependent on the number of fire sources. A different amount of smoke is formed in the room depending on the material of the objects, the ventilation parameters, and the tightness of the room;
7. The toxicity of burning products in a room, which determines the magnitude of the negative effects on humans;
8. The humidity in a room primarily affects the initial state of the threshold value of the objects in the room.

Thus, the presented approaches to the analysis of the burning process allow its formalization in the form of a mathematical model, as well as the software implementation of the burning process in adaptive training complexes. The proposed method of graphical representation of the process of threshold values of virtual objects using the corresponding life cycle diagrams, a system of state transition rules that allow to simulate the processes of burning and extinguishing of any complexity and fully describe the life cycle of virtual reality objects.

## 3. Results

We combined the above approaches to the formalization of the processes of burning and extinction in the form of a mathematical model of changing the states of virtual objects CP as follows:

(1) $$CP\to \{o_{i}\},o_{i}\in O,$$

where $o_{i}\in O$—set of burning objects representing a three-dimensional model of a real object with its basic properties that directly affect only the burning process and, therefore, the modeling of this process.

**Figure 2:**
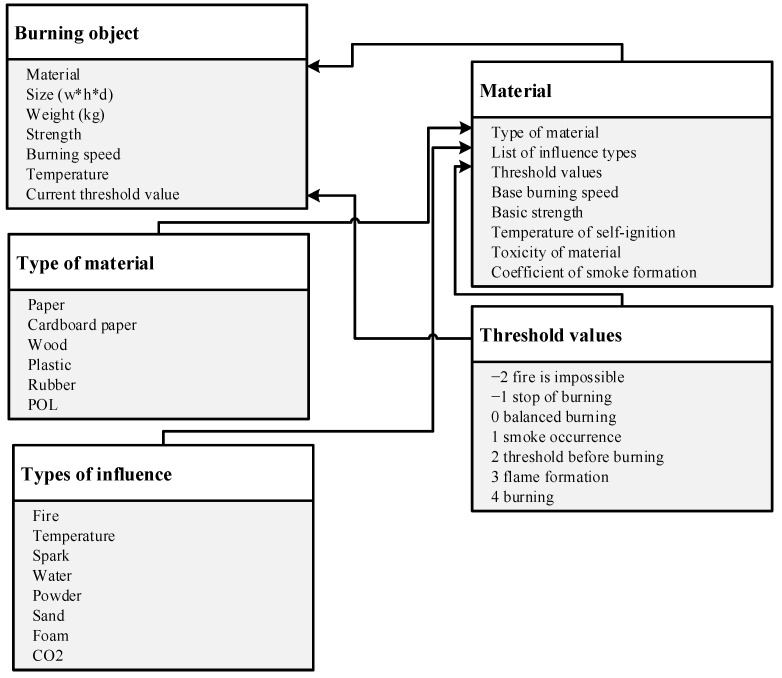
Structural diagram of the burning object. Source: developed by the authors.

We consider the basic properties of burning objects [16,20] and the associated processes of flame formation and extinguishing, presented in the diagram (Figure 2).

The main parameters of the object include the size $Size_{i}$, weight $Weight_{i}$ and type of material $TypeM_{i}$ from which this object is made, the strength of the object $H_{i}$, as well as a set of the following additional parameters: temperature $T_{i}$, current threshold value $S_{i}$ and burning speed $IS_{i}$. Then the following applies:

(2) $$o_{i}=(Size_{i},Weight_{i},TypeM_{i},H_{i},IS_{i},T_{i},S_{i}).$$

The strength of the object $H_{i}$ depends on the size, weight and type of material of the object. The following applies:

(3) $$(Size_{i},Weight_{i},TypeM_{i})\to H_{i}.$$

The strength of an object affects the time from the moment the object is ignited to its complete burning, when the object can no longer inflame.

The burning rate of an object is determined by the empirical function FS, dependent on the following parameters:

(4) $$IS_{i}=FS(S_{i},IS_{i},TypeM_{i}).$$

Also, the burning rate can change up or down within one state as a result of third-party effects (for example, quenching reduces speed and flame strengthening, on the contrary, increases).

Temperature $T_{i}$—the object’s own temperature, which also affects its environment. This parameter is determined by the function FT and depends both on the total environmental temperature $T_{E}$ and the temperature of near objects, as follows:

(5) $$T_{i}=FT(TE_{i},T_{i},\{T_{j}|j\neq i\}).$$

The burning rate depends directly on the current threshold value, and if the object is in fire, then the safety margin of the object will vary depending on the speed. The following applies:

(6) $$H_{i}=H_{i}-IS_{i}\cdot \Delta t,$$

where Δt—time of burning.

The main material of the object $TypeM_{i}$ also has a number of characteristics involved in the process of the combustion modeling, as follows:

The type of material is directly what the object consists of; for example, paper, cardboard, wood, plastic, rubber, petrol, oil and lubricants.

The types of influences are a list of impacts that can affect the state of the object.

The base burning speed and base strength act as coefficients in calculating the burning speed and strength of a particular object.

The self-ignition temperature is the temperature when an object can self-ignite.

The toxicity of the material during burning affects the state of the respiratory system of the ATC user, if you do not use protection means. As well as toxicity, the coefficient of smoke formation affects the state of the respiratory condition and the visibility of the ATC user.

The threshold conditions of the object $S_{i}$ replace each other continuously with a certain step, thus, you can determine the current state of the object in the range between two threshold values by the following formula:

(7) $$S_{i}=S_{i}+k\cdot \Delta t\cdot \frac{\left|T_{i}-T_{j}\right|}{T_{i}},$$

where k—coefficient determining the direction and intensity of changes in the threshold value of the object. It accepts values in the interval {−1;1}; however, for some materials (for example, gasoline) k large values {1000;1000} are used to pass certain threshold values in the shortest possible time; Δt—change time of threshold state; $T_{i},T_{j}$—the temperature of the object and the external environment (influence), respectively.

In addition to the parameters of a virtual object, the process of burning is significantly affected by environmental characteristics PE and a set of influences SE.

The characteristics of the virtual surrounding determine the volume of the room $Q_{E}$, ventilation parameters $V_{E}$, electricity $E_{E}$, hermetic seal condition $HR_{E}$, oxygen amount $OX_{E}$, smoke $SM_{E}$, toxicity $TX_{E}$, temperature $T_{E}$ and humidity $HM_{E}$ in the room, as follows:

(8) $$PE=(Q_{E},V_{E},E_{E},HR_{E},OX_{E},SM_{E},TX_{E},T_{E},HM_{E}).$$

The influences (events) $SE=\{se_{j}\}$ are aimed at changing the characteristics of objects and environments of virtual reality when the burning process moves either in a positive direction (burning, heating) or in a negative direction (extinguishing, cooling). The main parameters of influence include the following:

(9) $$se_{j}=(\lambda_{j},T_{j}^{se},t_{j}),$$

where $\lambda_{j}$—coefficient determining the intensity factor; $T_{j}^{se}$—temperature of impact; and $t_{j}$—total influence time including time intervals $t_{j,k}$, corresponding to different threshold values of the observed object.

The change in temperature of an object depends on the following factors:

Object burning, as follows:

(10) $$T_{i}=T_{i}+\lambda_{j}\cdot \frac{H_{i}}{H_{0}}\cdot t_{j}\cdot (T_{j}^{se}-T_{i}),$$

Object firefighting, as follows:

(11) $$T_{i}=T_{i}-\lambda_{j}\cdot \frac{H_{i}}{H_{0}}\cdot t_{j}\cdot (T_{i}-T_{j}^{se}),$$

Environmental influence, as follows:

(12) $$T_{i}=T_{i}+\lambda_{E}\cdot t_{E}\cdot (T_{i}-T_{E}),$$

where $\lambda_{E}$—coefficient that determines the direction and intensity of the influence of the environment, depending on the parameters of the environment, takes a positive sign if the object is colder than the environment and a negative otherwise; $t_{E}$—environmental influence time; and $H_{i}$ and $H_{0}$—the current and initial strength of the object, respectively.

The temperature is calculated at each time interval from one threshold to another by the formulas (10)–(12).

Using the above mathematical model of changing the states of virtual objects, the apparatus of threshold diagrams and rules for changing states, we proceed to the formalization of the burning process of virtual reality objects in ATC. As an example, we consider the simplified scenario for the ignition of an object made of wood as the main material and extinguishing it with water.

In order to formalize the process of changing events within the virtual reality, in addition to diagrams of the life cycle of virtual objects it is proposed to use a matrix representation that includes all attributes of the objects, surrounding space, sources of fire, etc. We formalize the change in the states of the objects in the process of burning and extinguishing, adding and subtracting the corresponding rows of matrices, estimating the obtained values of attributes

For a better understanding and reduction in the analytical calculations as part of this article, we introduce the following assumption: the calculation of temperatures is carried out discretely for each threshold value, and not continuously at each time interval. This assumption is removed during the software implementation of the mathematical model. The second assumption is as follows: this article does not provide calculations according to Formula (6), since in software the coefficients k are set empirically for each transition from one threshold state to another and presenting these calculation formulas under the considered examples does not make sense. The third assumption is as follows: we accept the following abbreviation to reduce the volume of calculation formulas:

(13) T=100→T=200≡T=100→200.

The scenario is to simulate the flame formation process of an object with periodic exposure to open fire with subsequent liquidation of fire.

**Figure 3:**
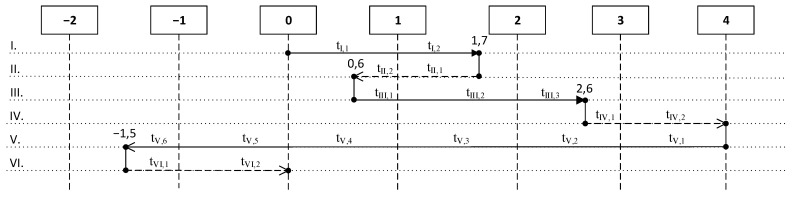
Diagram of the life cycle of object in the first scenario. Source: developed by the authors.

The diagram in Figure 3 displays the life cycle of an object during the first scenario at the flame formation and subsequent liquidation of fire. The duration of each threshold state within an event is indicated by $t_{j,k}$.

Initially, the observed object o is in the neutral threshold state «0». The state of the environment PE is also conditionally neutral, as follows:

(14) $$o+PE=\left[\begin{array}{l} H=5000\\ S=0\\ T=25\\ IS=0 \end{array}\right]+\left[\begin{array}{l} \lambda_{E}=0.02\\ T_{E}=25 \end{array}\right]=\left[\begin{array}{l} H=5000\\ S=0\\ T=25\\ IS=0 \end{array}\right].$$

At a certain point in time an event occurs $se_{I}$—direct exposure of an open flame to an observable object during $t_{I}$ = 5 s before the event $se_{II}$. During the exposure time, the object reaches the state “1” in 3 s, after which smoke begins to appear and reaches the intermediate state “1.7” in 2 s.

(15) $$o+se_{I}+PE=\left[\begin{array}{l} H=5000\\ S=0\\ T=25\\ IS=0 \end{array}\right]+\left[\begin{array}{l} \lambda_{I}=0.05\\ T_{I}^{se}=500\\ t_{I}=5 \end{array}\right]+\left[\begin{array}{l} \lambda_{E}=0.02\\ T_{E}=25\\ t_{E}=2 \end{array}\right]=\left[\begin{array}{l} H=5000\\ S=1.7\\ T=120\\ IS=0 \end{array}\right],$$

(16) $$t_{I}=\left\{ \begin{array}{l} t_{I,1}=3,S=0\to 1,T=25\to 100,H=5000;\\ t_{I,2}=2,S=1\to 1.7,T=100\to 137,H=5000. \end{array}\right.$$

We noted that at the stage $t_{I,2}$, the environment already influences because of the temperature difference between the object and the environment. After the end of the influence according to the rules of the transition states, an object from the intermediate state “1.7” will tend to a threshold value “0” under the influence of a neutral environment. Thus, an event $se_{II}=PE$ begins—the end of exposure to an open flame on an object during $t_{II}$ = 30 s (cooling process).

(17) $$o+PE=\left[\begin{array}{l} H=5000\\ S=1.7\\ T=137\\ IS=0 \end{array}\right]+\left[\begin{array}{l} \lambda_{E}=0.02\\ T_{E}=25\\ t_{E}=30 \end{array}\right]=\left[\begin{array}{l} H=5000\\ S=0.6\\ T=80\\ IS=0 \end{array}\right],$$

(18) $$t_{II}=\left\{ \begin{array}{l} t_{II,1}=16,S=1.7\to 1,T=137\to 100,H=5000\to 5000;\\ t_{II,2}=14,S=1\to 0.6,T=100\to 80,H=5000\to 5000; \end{array}\right..$$

The next event $se_{III}$—continuation of exposure to open flame, similarly $se_{I}$, during $t_{III}$ = 14 s, as follows:

(19) $$\begin{array}{l} o+se_{III}+PE=\left[\begin{array}{l} H=5000\\ S=0.6\\ T=80\\ IS=0 \end{array}\right]+\left[\begin{array}{l} \lambda_{III}=0.05\\ T_{III}^{se}=500\\ t_{III}=14 \end{array}\right]+\left[\begin{array}{l} \lambda_{E}=0.02\\ T_{E}=25\\ t_{E}=14 \end{array}\right]=\\ =\left[\begin{array}{l} H=4737\\ S=2.6\\ T=310\\ IS=25 \end{array}\right],\left[\begin{array}{l} \lambda_{E}=0.01\\ T_{E}=40 \end{array}\right], \end{array}$$

(20) $$t_{III}=\left\{ \begin{array}{l} t_{III,1}=1,S=0.6\to 1,T=80\to 100,H=5000\to 5000;\\ t_{III,2}=2.5,S=1\to 2,T=100\to 150,H=5000\to 5000;\\ t_{III,3}=10.5,S=2\to 2.6,T=150\to 310,H=5000\to 4737.5. \end{array}\right.$$

Since the object crossed the threshold value “2”, the burning process becomes irreversible even without an external source of flame (in this case, it is considered that the object itself becomes a source of flame for itself). The temperature of the object also begins to affect the ambient temperature and the degree of its influence on the object. This is reflected in the event $se_{IV}$, occurring for $t_{IV}$ = 20 s, as follows:

(21) $$o+se_{IV}+PE=\left[\begin{array}{l} H=4737\\ S=2.6\\ T=310\\ IS=25 \end{array}\right]+\left[\begin{array}{l} \lambda_{IV}=0.05\\ T_{IV}^{se}=500\\ t_{IV}=20 \end{array}\right]+\left[\begin{array}{l} \lambda_{E}=0.01\\ T_{E}=40\\ t_{E}=20 \end{array}\right]=\left[\begin{array}{l} H=4400\\ S=4\\ T=390\\ IS=50 \end{array}\right],$$

(22) $$t_{IV}=\left\{ \begin{array}{l} t_{IV,1}=11,S=2.6\to 3,T=310\to 380,H=4737.5\to 4460;\\ t_{IV,2}=9,S=3\to 4,T=380\to 390,\\ IS=25\to 50,H=4460\to 4400. \end{array}\right.$$

The next event $se_{V}$— a person’s influence in order to stop the fire using water for $t_{V}$ = 49 s, as follows:

(23) $$o+se_{V}+PE=\left[\begin{array}{l} H=4400\\ S=4\\ T=390\\ IS=50 \end{array}\right]+\left[\begin{array}{l} \lambda_{V}=-0.05\\ T_{V}^{se}=10\\ t_{V}=49 \end{array}\right]+\left[\begin{array}{l} \lambda_{E}=0.01\\ T_{E}=40\\ t_{E}=49 \end{array}\right]=\left[\begin{array}{l} H=3420\\ S=-1.5\\ T=70\\ IS=0 \end{array}\right],$$

(24) $$t_{V}=\left\{ \begin{array}{l} t_{V,1}=6.5,S=4\to 3,T=390\to 300,\\ IS=50\to 25,H=4400\to 4070;\\ t_{V,2}=7,S=3\to 2,T=300\to 230,H=4070\to 3890;\\ t_{V,3}=10,S=2\to 1,T=230\to 160,H=3890\to 3640;\\ t_{V,4}=8.5,S=1\to 0,T=160\to 120,\\ IS=25\to 0,H=3640\to 3420;\\ t_{V,5}=9,S=0\to -1,T=120\to 90,H=3420;\\ t_{V,6}=8,S=-1\to 1,T=90\to 70,H=3420. \end{array}\right.$$

The change in the state of the object occurs up to the threshold value “−1.5” during the time $t_{V}$, when the burning of the object stops and re-ignition without external influence is impossible.

At the point “−1.5” the water supply stops and, accordingly, its influence—an event occurs $se_{VI}=PE$. According to the rule of the object’s tendency to the neutral state “0” after the stop of water for time $t_{VI}$ = 108 s under the influence of the environment only, the object changes to the state “0”, in which it remains until the onset of new events.

(25) $$o+PE=\left[\begin{array}{l} H=3420\\ S=-1.5\\ T=70\\ IS=0 \end{array}\right]+\left[\begin{array}{l} \lambda_{E}=0.01\\ T_{E}=40\\ t_{E}=108 \end{array}\right]=\left[\begin{array}{l} H=3420\\ S=0\\ T=25\\ IS=0 \end{array}\right],\left[\begin{array}{l} \lambda_{E}=0.02\\ T_{E}=30 \end{array}\right]$$

(26) $$t_{VI}=\left\{ \begin{array}{l} t_{VI,1}=66,S=-1.5\to -1,T=70\to 50,H=3420;\\ t_{VI,2}=62,S=-1\to 0,T=50\to 25,H=3420. \end{array}\right.$$

**Figure 4:**
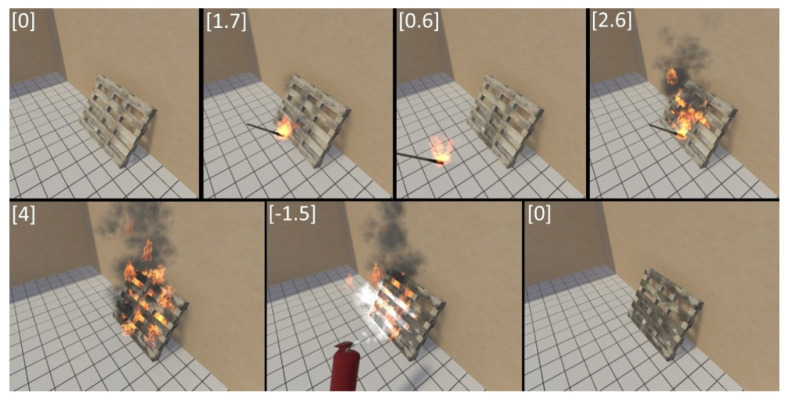
Software implementation of the burning process. Source: developed by the authors in Unity3d.

Further, on the basis of the presented formalization, a software implementation is carried out in the C # programming language in the Unity3d (Unity Technologies, San Francisco, CA, USA) development environment [21]. When developing a mathematical model, we took into account the object-oriented paradigm used in Unity3d, the transfer of the structure of objects and burning processes into the program code is quite fast and simple. An example of the burning process in the considered scenario is presented in Figure 4.

The proposed approach made it possible to render the combustion process at 60 frames per second (FPS) for the HTC Vive (HTC Corporation, Xindian, New Taipei, Taiwan) virtual reality helmet (two screens with a resolution of 1920 × 1080 pixels) on a computer equipped with an Nvidia GTX 1070 (NVIDIA Corporation, Santa Clara, CA, USA) video card (floating point performance of 6.463 gflops), an Intel Core i7 processor (Intel Corporation, Santa Clara, CA, USA), and 32 GB of RAM. The increase in the number of objects did not affect the performance. The implementation of the deterministic fire model in the test scene led to a significant drop in performance, on a simple object in an empty room, it was impossible to reach 60 FPS, which is the minimum comfortable value for a virtual reality helmet. A preliminary assessment of the complexity of the project using the COCOMO II methodology also showed that the use of a deterministic model of fire propagation with the need for additional optimization of the project will lead to an increase in the complexity and timing of the implementation of the virtual simulator by 2–3 times, which will directly affect its cost.

The universality of the presented approaches allows simulating the burning processes of objects of various shapes, materials and sizes, as well as, regardless of the parameters of the room, to successfully carry out realistic visualization of the fire and extinguishment of objects.

## 4. Discussion

The training of personnel for activities in emergency situations is an important and urgent task, since the safety of people and industrial facilities directly depends on this [4,6]. One of the effective training tools is the adaptive training complex, and the quality and speed of training on them depends on the degree of involvement in virtual reality in the process of training in ATC [4,6,21,22]. Therefore, an important task in the development of ATC is the reliable and high-quality visualization of physical processes, especially the spread of fire. In addition to direct visualization, the correct computer simulation of the spread of fires allows us to solve related problems, among which the definition of the evacuation time during fires and the negative impact on humans [1,2], the issues of effective forest fire control [3] should be noted.

However, it is impossible to simulate the burning processes without adequate software [23]. The article [18], for example, presents a method for modeling and visualizing the spread of smoke and fires, based on the initial solution of the equations for the flow of gas and liquid in a two-dimensional formulation. In the article [19], the methods of using empirical data on fires and burning simulation results based on the gas dynamics equations in virtual reality simulators are considered. In the work [20], the issues of burning modeling are considered in detail. The method proposed in this article is based on the physical modeling of laminar and turbulent fire. The obtained results allow simulating the spread of fire from evaporating combustible liquids and solid combustible substances.

A common drawback of most of the work is the need to solve differential equations of the mathematical model of burning (or the analysis of a larger amount of empirical data). Therefore, their use in the direct implementation of fire spread in virtual reality leads to a significant timing for calculations and solving differential equations and, consequently, an increase in the cost of the training complex and a decrease in its performance. Therefore, an urgent task is the development of a mathematical model adapted for application in visualization tools and ensuring the correctness of the calculations of the burning processes in ATC. Naturally, such a model will be simplified and contain a number of assumptions; however, within the framework of the task, it is only necessary to ensure visual correspondence of the simulated burning processes to real ones.

Further studies will focus on eliminating all the accepted assumptions in analytical calculations, improving the accuracy of the mathematical model under consideration by including environmental parameters, air flows characteristics into the formulas, and considering the simultaneous extinguishing and burning of objects. This will improve the accuracy of the results, which will also affect the accuracy of the visualization of virtual objects and processes during their software implementation.

Thus, in comparison with the existing scientific research, the work was carried out on the formalization of the burning processes of virtual reality objects within the framework of this article. Using the apparatus of set theory and graph theory, a mathematical model of the burning process is presented, as well as a diagram of the threshold values of the objects and the rules of their states change. The conducted studies allowed successfully applying the software implementation of the burning process within the Unity3d development sphere in the design of ATC for ergatic systems for professional purposes [24].

## 5. Conclusions

The issue of training personnel for activities in emergency situations remains relevant. Adaptive training complexes are one of the effective tools for solving this problem, allowing, due to the interactivity and realism of the processes modeled in the virtual space, to provide the necessary speed and efficiency of training.

In this article, an important aspect of ATC design is considered—the formalization of physical processes in virtual space using the example of burning objects. This process was sufficiently studied; however, the existing mathematical models are not adapted for use in training complexes, leading to a significant increase in the cost of ATC and a decrease in performance due to the complexity of the calculations. Therefore, an adapted mathematical model is proposed that allows us to formalize the structure of burning objects, their basic properties and the processes of changing states, starting from the flame formation in an object and ending with their complete destruction or extinguishment.

In addition, in order to solve the problem of formalizing the burning process, we offer original diagrams of the life cycle of virtual objects and a system of rules for changing their states, which allow tracing the entire life cycle of objects and the procedure for changing states. The patterns of state transitions are formalized using the matrix representation of the objects’ properties. This approach is distinguished by its versatility and can be used to model other physical processes (smoke, flooding, freezing, spread of toxic gases, etc.). Thus, the conducted research can be used to formalize the physical processes in the training complexes for ergatic systems for professional purposes.

## Figures and Tables

**Table 1:** Threshold values of burning process.

Threshold Number	Description and Condition of Occurrence
«−2»	The condition of the object when the fire is absolutely impossible. This condition is achieved after a strong impact on the object with water, foam or other substance that prevents burning.
«−1»	The formal boundary between such states of the object as “wet” and “dry”.
«0»	The condition describing an object under normal conditions. The object tends to this value in the absence of various impacts. Also, after complete destruction (burning), the object returns to this state.
«1»	The initial stage of flame formation of the object. When it is achieved, only visualization of smoke occurs, without an open flame and changing the color of the object.
«2»	The formal boundary, at the crossing of which the object will no longer tend to its normal state (threshold value 0).
«3»	When reaching this value, an open flame, smokes and color changes of the object take place.
«4»	The maximum degree of object burning at which the parameters of the height of the flames, smoke and temperature effects have the maximum value.

Source: developed by the authors at the stage of system analysis.
